# In-vitro effects of the antimicrobial peptide Ala8,13,18-magainin II amide on isolated human first trimester villous trophoblast cells

**DOI:** 10.1186/1477-7827-9-49

**Published:** 2011-04-16

**Authors:** Jayasree Sengupta, Meraj Alam Khan, Berthold Huppertz, Debabrata Ghosh

**Affiliations:** 1 Department of Physiology, All India Institute of Medical Sciences, New Delhi, India; 2 Institute of Cell Biology, Histology and Embryology, Medical University Graz, Austria

## Abstract

**Background:**

Research on antimicrobial cationic peptides (AMPs) has gained pace toward using their potential to replace conventional antibiotics. These peptides preferentially interact with negatively charged membrane lipids typically seen in bacteria and thereby lead to membrane perturbations and membrane dysfunction. However, one possible disadvantage of AMP drugs is their potential for toxicity, especially to those cells which display externalization of negatively charged moieties to the outer leaflet of the plasma membrane during the process of syncytialization. Human placental villous trophoblast is one such cell type. Indeed, intra-vaginal administration of an antimicrobial cationic peptide Ala8,13,18-magainin II amide (AMA) which is a synthetic analogue of magainin 2 derived from *Xenopus *frog has been observed to result in inhibition of pregnancy establishment in monkeys. However, only little is known about the cellular behavior of early placental cytotrophoblasts (CTB) in the presence of cationic antimicrobial peptides. It is believed that suitable cell culture approaches using AMA as a representative alpha-helical AMP may yield tangible knowledge in this regard.

**Methods:**

Immunocytochemical (ICC) analyses using confocal microscopy (n = 6 for each treatment sub-group) and Western blot (WB) method (n = 5 for each treatment sub-group) of CTB differentiation based on synthesis of beta-hCG and hPL, and apoptosis based on apoptosis-associated cytokeratin 18 neo-epitope (CK18f) were performed for CTB isolated from human first trimester placental villi and grown in serum-free primary culture for 24 h, 48 h and 96 h on rat-tail collagen with and without AMA (1000 ng/ml). Moreover, secretion of beta-hCG and hPL into conditioned media from isolated CTB grown *in vitro *for 24 h, 48 h and 96 h (n = 6/each sub-group) with and without AMA was examined using enzyme immunoassays. Furthermore, TUNEL assay, and cell viability based on LDH leakage into medium (n = 6/each sub-group) were assessed to examine the phenomenon of cell death with time and administration of AMA.

**Results:**

CTB in serum-free primary culture showed increased (P < 0.05) level of synthesis and secretion of beta-hCG and hPL with time, and higher (P < 0.05) level of cellular cytokeratin 18 neo-epitope and number of TUNEL-positive cells, and LDH activity in conditioned medium at 96 h of culture. Exposure of CTB to AMA resulted in lower (P < 0.05) level of synthesis and secretion of beta-hCG and hPL, as well as, an increase (P < 0.05) of cellular cytokeratin 18 neo-epitope and number of TUNEL-positive cells, and LDH activity in conditioned medium at 96 h as compared to the control treatment.

**Conclusions:**

Administration of AMA resulted in attenuation of differentiation, enhancement in apoptosis and loss of viability in early placental villi trophoblast cells in primary culture. Thus, it appears that administration of alpha-helical AMP may adversely affect the process of placentation and pregnancy outcome.

## Background

One major challenge of medicine today is the growing number of bacterial strains resistant to conventional antibiotic therapies. Hence, the need for new antibiotics or even alternative compounds has stimulated research in the field of antimicrobial peptides to be used as human therapeutics [1,2]. To this effect, research on gene-encoded cationic antimicrobial peptides (AMPs) has gained pace in the recent time [3]. AMPs can be defined as being short peptides (10-50 amino acids) with an overall positive charge (+2 to +9) and a substantial proportion of (>30%) of hydrophobic residues [3-5]. These chemical properties in AMPs generally result in folds into amphiphilic structures, especially upon contact with membranes and give rise to formation of separate patches rich in positively charged and hydrophobic amino acids [3-5]. There are four broad structural groups of AMPs: α-helical peptides (for example, cercopin B, magainins, LL37), extended structures rich in glycine, proline, tryptophan, arginine, histidine (for example, indolicidin and histatin 1), peptides with one disulfide bond (for example, bactenecin and esculentin A), and β-sheet peptides stabilized by two or more disulfide bridges (for example, human defensins and protegrins) [3-6]. These peptides preferentially interact with negatively charged lipids, which are major components of bacterial cell membranes resulting in membrane perturbations such as pore formation, alterations of the curvature strain and induction of lipid-peptide domain formation [7]. Such perturbations may alter the micro-environment of membrane proteins resulting in membrane dysfunction. In mammalian cell membranes however negatively charged lipids such as phosphatidylserine are mostly located in the inner leaflet of the membrane and thus are not exposed to the outer surface of the cell. However, during pregnancy such peptides may jeopardize placental villous trophoblasts because these cells show externalization of negatively charged phosphatidylserine moieties to the outer leaflet of the plasma membrane during the process of syncytialization [8]. This may make them vulnerable to AMPs [9]. In fact, one possible disadvantage AMP drugs, especially for α-helical AMPs, is their potential for toxicity [10], although the issue of AMP drugs mediated toxicity on placental trophoblasts has hitherto been little addressed [3].

Ala^8,13,18^-magainin II amide (AMA) is a synthetic AMP [11,12] belonging to α-helical peptide group of AMPs and modified from natural magainin 2 obtained from African frog *Xenopus leavis *[13,14]. Replacement of three amino acid residues (Ser^8^, Gly^13^, Gly^18^) of magainin 2 peptide with alanine enhances its antimicrobial activity, and amidation of its terminal amino acid renders stabilization to its α-helical conformation [12]. It shows chemical properties that are very similar to that of human α-helical AMP, cathelicidin peptide LL-37 [3,6] and is commercially available. In a previous study, we have reported that intra-vaginal administration of AMA resulted in the inhibition of blastocyst implantation in the rhesus monkey [9]. Further study in the same species indicated that the anti-nidatory effect of AMA might be resulted from its inhibitory effect on trophoblast cell differentiation [15]. However, little is known about the cellular behavior of early placental trophoblast cells in the presence of cationic antimicrobial peptides. It is assumed that suitable cell culture approaches using AMA as an AMP of non-defensins group may yield tangible knowledge in this regard.

In the present study, we aimed to examine the *in vitro *effects of AMA on cellular processes like differentiation and apoptosis in primary human placental villous cytotrophoblasts (CTB) isolated from first trimester placental tissues. To this end, we have employed well known markers of cellular phenotype for trophoblast differentiation, apoptosis and viability in primary cell culture system [16]. Table 1 provides a summary of the target proteins examined in the present study. The assessment of CTB differentiation in culture has been performed by examining synthesis and secretion of human chorionic gonadotropin beta (βhCG) and human placental lactogen (hPL) [16-18]. The assessment of apoptosis was done by examining the level of caspase-mediated cleavage of cytokeratin 18 yielding a specific neo-epitope (cytokeratin 18 neo-epitope, CK18f), and nuclear DNA fragmentation using terminal deoxynucleotidyl transferase (TdT) enzyme based TUNEL (TdT dUTP nick end labeling) assay in trophoblast cells. Both methods are based on well characterized phenomena of apoptosis at cytoplasm and nucleus, respectively; these are considered robust approaches in studying apoptosis [19,20]. The loss of cell viability in culture with and without AMA at different time points was assessed from lactate dehydrogenase (LDH) activity in the conditioned media. This approach is based on the fact that the loss of intracellular LDH into the culture medium is a reliable indicator of irreversible cell membrane damage and cell death [21].

**Table 1:** Characteristics of targets and primary antibodies used in the study

Antigen	Specification of antibody	Final concentration	Purpose*
β-actin	Sheep IgG^a^	2.0 μg/ml	Internal normalization control, used in WB
Cytokeratin 7	Mouse IgG^b^	6 μg/ml	Epithelial cell marker, used in ICC
Cytokeratin 18 neo-epitope (CK18f)	Mouse IgG^c^	1:50 (WB); 1:20 (ICC)	Early apoptosis marker, used in WB and ICC
βhCG	Rabbit IgG^c^	4 μg/ml (WB); 10 μg/ml (ICC)	CTB differentiation marker, used in WB and ICC
hPL Goat IgG^d^		3 μg/ml (WB); 7 μg/ml (ICC)	CTB differentiation marker, used in WB and ICC
Vimentin	Mouse IgG^a^	3 μg/ml	Cytoskeletal protein marker for fibroblasts, used in ICC
Vitronectin receptor, Integrin αVβ3 (CD51)	Mouse IgG^e^	10 μg/ml	Invasive CTB marker, used in ICC
von Willebrand factor	Rabbit IgG^c^	25 μg/ml	Endothelial cell marker (in Weibel-Palade bodies), used in ICC

*see references [17-19,23-27,32] for details.

CTB, cytotrophoblast cells. ICC, immunocytochemistry. WB, Western blot analysis.

^a^R&D Systems, Minneapolis. ^b^Sigma Chemical, St Louis, MO, USA. ^c^Dako, Glostrup, Denmark. ^d^Santa Cruz Biotechnology, Santa Cruz, CA, USA. ^e^Zymed Laboratories, San Francisco, CA, USA.

## Methods

### Tissue samples and chemicals

Human placental samples (*N *= 47) were obtained from women (age: 23-37 years) undergoing elective surgical termination of singleton pregnancy between 6 and 8 weeks of gestation (timed from last menstrual period) without undergoing any prior medication. All women provided their written informed consent. The Ethics Committee of the All India Institute of Medical Sciences approved the research study. The study also complied with the Helsinki declaration. Placental samples were collected in sterile ice-cold phosphate buffered saline (PBS, pH 7.4) and transported on ice to the laboratory within 15 min after collection for further processing. All the chemicals were obtained from Sigma Chemical Co. (St. Louis, MO, USA), if not stated otherwise.

### Isolation of placental villous trophoblast cells

CTB were isolated from freshly collected first trimester placental villi as described previously [22-26]. Briefly, villous tissues (2-3 g) were dissected from chorionic membranes and washed with sterile cold Ca^2+^/Mg^2+ ^free PBS (pH 7.4) containing gentamycin (50 μg/ml) and D-glucose (1 mg/ml). Placental tissues were incubated in an enzyme mixture [0.25% (w/v) trypsin, 0.02% (w/v) deoxyribonuclease type-I (DNase I), 15 mM HEPES, 5 mM magnesium sulphate, penicillin (100 IU/ml), streptomycin (100 μg/ml) and amphotericin B (2.5 μg/ml)] at 37°C initially for 30 min and then for another three cycles of 10 min each. Cell suspension was passed through a pre-equilibrated mesh filter (pore size 60 μm) to remove cellular debris. The filtrate was subjected to enrichment on a preformed 10% to 70% Percoll gradient at 800 × g for 20 min at 20°C. The mononuclear cells were immunopurified by depletion of CD45-positive leucocytes using MACS microbeads conjugated with monoclonal mouse antibody against CD45 and magnetic separation columns type LS in combination with a MidiMACS separator (Miltenyi Biotec, Bergisch Gladbach, Germany) [25]. The negative fraction containing an enriched villous trophoblast cell population was collected and immunocharacterized for cytokeratin 7, βhCG, CD51, von Willebrand factor (vWF) and vimentin.

### Cell culture

The methodological details of CTB culture have been detailed previously [24-26]. Briefly, isolated mononucleated CTB were plated at a density of 1 × 10^5^/cm^2 ^on collagen I and cultured at 37°C in a humidified air atmosphere of 5% CO_2 _and in complete medium [DMEM: F12 (1:1), 10% (v/v) fetal calf serum, penicillin (100 IU/ml), streptomycin (100 μg/ml), amphotericin B (2.5 μg/ml)] for 24 h to allow for their attachment to collagen. Subsequently, the cells were maintained by daily feeding in serum-free medium with antibiotics and antimycotics as described above and supplemented with insulin (5 μg/ml), transferrin (5 μg/ml), selenium (5 ng/ml) and hydrocortisone (0.5 μg/ml) based on the initial optimization observation that addition of these factors render more number of target cells attached, viable and aggregated in serum-free primary culture on rat-tail collagen. It has also been earlier reported that collagen and insulin supported CTB differentiation in primary culture [27]. The same pools of cells were treated with or without AMA (1000 ng/ml) in triplicates to harvest at 24 h, 48 h and 96 h. The concentration of AMA in the present study was selected based on the previously reported dose finding study on viability, hCG secretion and invasion efficiency for isolated CTB indicating that AMA at 1000 ng/ml affected trophoblast invasion into collagen coated membrane with 8 μm pores without any marked effect on hCG secretion and mitochondrial viability at 48 h in presence of serum containing medium [25]. In initial experiments, method optimization for the methods employed in the present study was performed at three time points as mentioned above in at least three successive satisfactory experiments.

### TUNEL assay

After termination of cultures at 24 h (*n = 6*), 48 h (*n = 6*) and 96 h (*n = 6*) in triplicates, cells treated with and without AMA (1000 ng/ml) were subjected to *in situ *detection of nuclear fragmentation according to the methods described elsewhere [28,29]. In brief, cells were washed in phosphate buffer saline (PBS, pH 7.4), fixed in 4% (w/v) paraformaldehyde in PBS followed by permeabilization, quenching and incorporation of labeled nucleotides (BrdU) with TdT enzyme reaction onto free 3'OH ends of DNA fragments and their detection with the help of biotinylated anti-BrdU antibody using TACS-XL kit obtained from Trevigen, Inc. (Gaithersburg, MD, USA). Samples along with positive control with nuclease treatment and negative control without enzyme were run as per the method protocol provided by the supplier. The labeled cells were counterstained with nuclear fast red and cell numbers were estimated from nuclear counts. All samples were analysed in one run.

### Immunofluorescent staining and image analysis

After termination of culture at 24 h, 48 h, and 96 h, cells treated with and without AMA (1000 ng/ml) were subjected to immunocytochemical (ICC) staining using various antibodies as described in Table 1. Immunofluorescent staining was performed on six samples in duplicates for each treatment group using a method described earlier [30]. Appropriate fluorochrome-conjugated secondary antibodies (Molecular Probes, Grand Island, NY, USA) were used for visualization. Specificity of the antibody binding was assessed by omitting primary antibodies, immunoadsorption of primary antibodies with target antigens, replacing primary antibodies with unrelated IgG from same species and other species, omitting secondary antibodies, and replacing secondary antibodies with unrelated IgGs from same or other species in parallel cultures.

Non-overlapping images (at least eight) from entire area of confluence of each culture after ICC were examined using a Confocal laser scanning microscope (Leica Microsystems, Wetzlar GmbH, Germany). The cells were detected using an interactive planimeter analyzer only in cases where discernibility was distinct. The immunopositive cells were identified by detecting positive profiles in digitized images. Following image grabbing and segmentation, digitalized images were subjected to image analysis for estimation of immunopositive area fraction (in per cent) based on manual outlining method and using an optimized grey level threshold yielding normal distribution of image pixels throughout the entire gray scale and shading correction. Pixel calibration was done against the standard provided by the manufacturer. The per cent immunostained areas was measured using a precalibrated computer-assisted digital image analysis system (Leica QWIN DC 200, Cambridge, UK) as described elsewhere [30]. The nuclei were counterstained with diamidino-6-phenylindole (DAPI) (Molecular Probes), and cell numbers were estimated from DAPI positive nuclear counts.

### Immunoblots

After termination of cultures at 24 h (*n = 5*), 48 h (*n = 5*) and 96 h (*n = 5*) in duplicate with and without AMA (1000 ng/ml), cells were subjected to Western blot (WB) analysis. Profiles of the candidate proteins in cell homogenates were assessed in samples of 25 μg protein content along with pre-stained molecular weight markers based on SDS-PAGE/Western immunoblotting method on nitrocellulose membrane using electrophoresis and trans-blot equipment, and chemicals obtained from Bio-Rad (Hercules, CA, USA) as described elsewhere [30]. Final visualization was achieved by using Vectastain ABC immunoperoxidase kits (Vector Laboratories). Respective primary antibody and secondary antibody controls were run simultaneously to examine the specificity of procedure. The molecular weights and semi-quantitative densitometric analysis of bands were determined using a densitometric equipment (Pharos FX Plus Molecular Imager, Bio-Rad, Hercules, CA, USA) and an optimized densitometric analysis software (PD Quest Advanced, Bio-Rad). The integrated measures of optical densities for individual antigens were calculated from log of transmittance for each of the target antigen and normalized with that of β-actin.

### Analysis of βhCG, hPL and LDH in conditioned media

After termination of cultures at 24 h (*n = 6*), 48 h (*n = 6*) and 96 h (*n = 6*) in triplicates with and without AMA (1000 ng/ml), supernatant from each cell culture well was collected, centrifuged and stored at -80°C for estimation of secreted bhCG and hPL by enzyme immunoassays (25) and lactate dehydrogenase (LDH) activity using standardized methods. The immunoassay kits for βhCG and hPL were purchased from DRG International Inc. (New Jersey, NJ, USA). The ranges of detectable concentrations in the assays were 5 - 300 mIU/ml for βhCG and 1 - 20 mIU/ml for hPL. All samples were analysed in one run. The intra-assay coefficients of variation for both enzyme immunoassays were less than 9%.

The potential cytotoxic effect of AMA (1000 ng/ml) to isolated CTB in culture with or without AMA at different time points was assessed by lactate dehydrogenase (LDH) leakage into the conditioned culture medium using a LDH kit obtained from Sigma. The assay is based on the conversion of lactate to pyruvate in the presence of LDH with parallel reduction of NAD estimated at 340 nm in a Benchmark Plus Spectrophotometer (Bio-Rad, Hercules, CA, USA) with necessary corrections [31]. All samples were analysed in one run. The intra-assay coefficients of variation for both assays were less than 6%.

### Statistical analysis

Statistical analysis of data was performed using Kruskal-Wallis test followed by rank sums Wilcoxon test using SPSS v17 (Chicago, IL, USA). The probability level of P = 0.05 was taken as the limit of significance.

## Results

**Figure 1:**
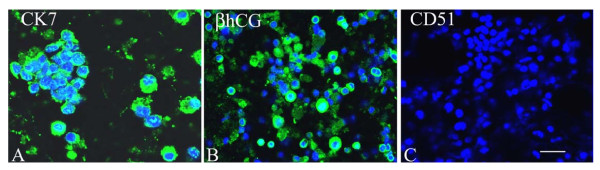
**Immunocytochemical characterization of isolated villous trophoblast cells**. Isolated cells were allowed to attach to the collagen biomatrix and immunostained for cytokeratin 7 (CK7; A; *green*) and βhCG (B; *green*) and vitronectin receptor (CD51; C; *green*). Nuclei are counterstained with DAPI (*blue*). *Bar *= 40 μm.

The cell yield was ~3 × 10^6 ^cells/g wet weight of placental villous tissue with cell viability in trypan blue exclusion method being more than 90%. These cells were consistently positive for cytokeratin 7 (94 ± 3%) and βhCG (93 ± 2%) and negative for vitronectin receptor (CD51; 92 ± 3%), vimentin and von Willebrand factor (99 ± 1%) in five cultures (Figure 1A-C).

### Effect of time course

**Figure 2:**
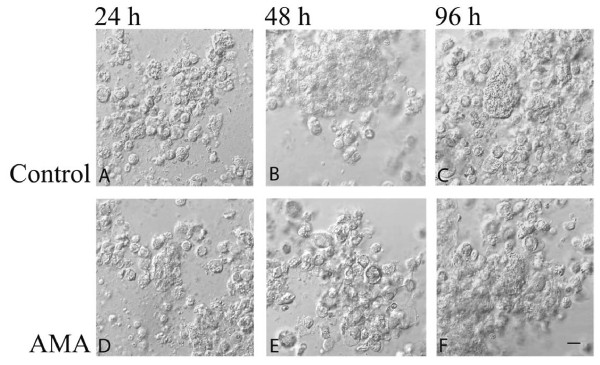
**Representative microphotography of isolated villous trophoblast cells grown on collagen biomatrix**. Isolated cells were allowed to attach to the collagen biomatrix and grow for 24 h (A, D), 48 h (B, E) and 96 h (C, F) with 1000 ng/ml AMA (D, E, F) and without AMA (A, B, C) in serum-free culture medium. *Bar *= 20 μm.

Isolated villous trophoblast cells grown on rat-tail type I collagen in serum-free medium yielded adherent spheroid cells at 24 h (Figure 2A), showed aggregations at 48 h (Figure 2B), and formed distinct aggregates of fused cells at 96 h (Figure 2C).

**Figure 3:**
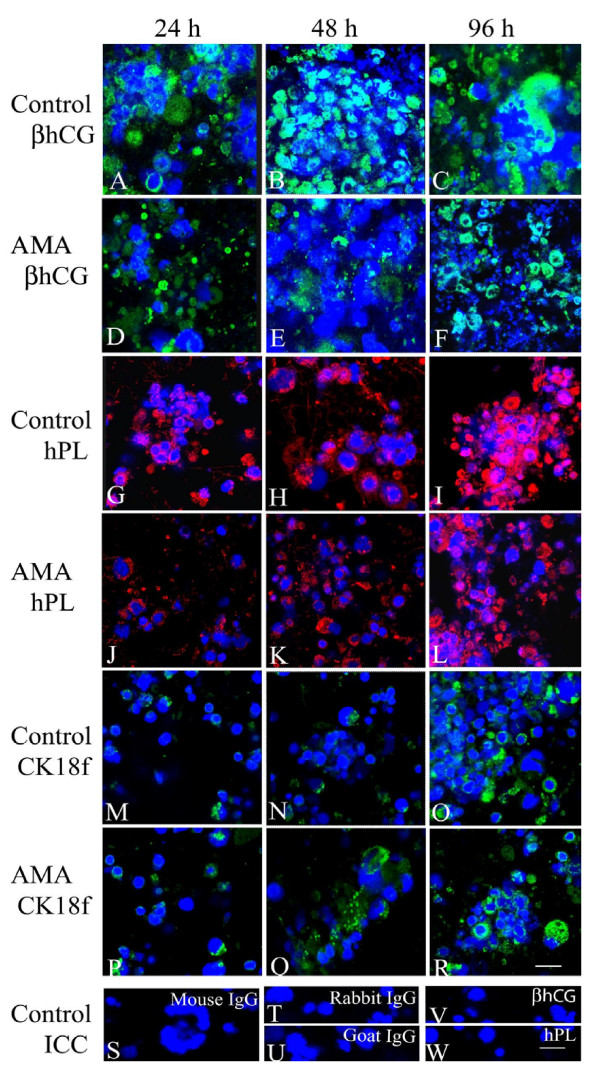
**Effect of time and magainin on immunopositive levels of βhCG, hPL and cytokeratin 18 neo-epitope (CK18f)**. Isolated villous trophoblast cells grown on collagen biomatrix for 24 h (A, D, G, J, M, P), 48 h (B, E, H, K, N, Q) and 96 h (C, F, I, L, O, R) show immunopositive staining for βhCG (A-F; *green*), hPL (G-L; *red*), and cytokeratin 18 neo-epitope (CK18f; M-R; *green*) treated with AMA (D-F, J-L, P-R) and without AMA(A-C, G-I, M-O). The replacement of primary antibody with non-immune mouse IgG (S), non-immune rabbit and goat IgGs (T, U), as well as, pre-neutralization of primary antibodies for βhCG (V) and (hPL) (W) with specific target antigens show no immunopositive staining. Nuclei are counterstained with DAPI (*blue*). *Bars *= 20 μm.

**Figure 4:**
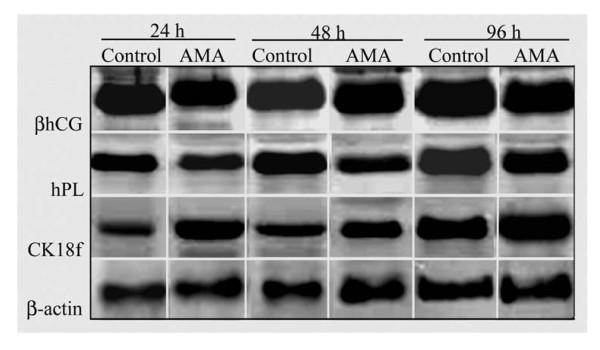
**Representative Western blot analysis of immunopositive levels of βhCG, hPL and cytokeratin 18 neo-epitope (CK18f) in cytotrophoblast cells grown in primary culture**. Cell lysates of isolated villous trophoblast cells grown on collagen biomatrix for 24 h, 48 h and 96 h with or without AMA (1000 ng/ml) were electrophoretically separated and subjected to immunoblot analysis using β-actin as the internal control.

Immunocytochemical analyses revealed that isolated primary villous cytokeratin 7- and βhCG-positive and vitronectin receptor (CD51)-negative CTB in culture synthesized an increasing amount of βhCG (P < 0.05) and hPL (P < 0.05) at 48 h as compared to 24 h, and at 96 h as compared to 24 h and 48 h (Table 2; Figures 3A-C, G-I). A concomitant increase (P < 0.05) in concentrations of βhCG and hPL was also detected in the conditioned media with time (Table 3). As shown in Figure 4 and Table 2, immunoblot analysis of βhCG and hPL protein expression after β-actin normalization in cell homogenates however detected significant change only at 96 h as compared to 24 h and 48 h with no change observed between 24 h and 48 h.

**Table 2:** Effect of AMA on time course characteristics of cellular behavior of placental villous CTB *in vitro*

*Test parameter*	Median value (ranges)		
	Time		
Treatment	24 h	48 h	96 h
**Specific stain positivity in ICC (n = 6/each treatment sub-group)**			
*βhCG*^a^			
Control	13.0 (8-18)	17.5* (11-27)	23.5^(*)^(18-32)
AMA	11.0 (9-20)	13.0 (9-22)	17.0 (11-24)
*hPL*^a^			
Control^1^	9.5 (7-12)	5.5* (9-20)	21.0^(*)^(14-30)
AMA	8.0 (6-13)	11.0 (8-18)	14.5 (10-21)
*Cyto18 neo-epitope*^a^			
Control	2.5 (1-4)	2.5 (1-5)	4.5^[*]^(3-8)
AMA	3.5 (2-6)	3.5 (2-7)	8.5^(*)^(5-11)
*TUNEL*^b^			
Control	0 (0-3)	0 (0-2)	6^[*]^(3-9)
AMA	3 (0-5)	3 (1-7)	11^(*)^(6-17)
**Optical density**c **in WB analysis (n = 5/each)**			
*βhCG*			
Control	20.5 (11-27)	22.0 (12-28)	30.5^(*)^(19-36)
AMA	18.0 (9-28)	21.5 (9-24)	22.5 (11-27)
*hPL*			
Control	9.5 (6-12)	10.0 (7-18)	16.5^(*)^
AMA	9.5 (7-13)	9.5 (6-18)	11.5 (9-21)
*Cyto18 neo-epitope*			
Control	4.0 (2-5)	4.0 (2-6)	6.5^[*]^(3-11)
AMA	5.5 (2-7)	5.5 (2-8)	9.5^(*)^(5-16)

^a^positive cellular area in per cent. ^b^number of positive cells in per cent. ^c^integrated optical density normalized by that of β-actin per 25 μg of protein.

^(*)^P < 0.05 compared with any other group; ^[*]^P < 0.05 compared with 24 h and 48 h groups in control treatment; *P < 0.05 compared with control treatment at 96 h.

**Table 3:** Effect of AMA on time course characteristics of levels of βhCG, hPL, and LDH activity in conditioned medium of placental villous CTB *in vitro*

*Test parameter*	Median value (ranges)		
	Time		
Treatment	24 h	48 h	96 h
*βhCG*^a^			
Control	10.5 (9-14)	21.0^[*] ^(13-27)	40.5^(*) ^(26-55)
AMA	8.5 (6-11)	14.0 (9-19)	25.5<*> (11-33)
*hPL*^a^			
Control	8.5 (7-11)	13.5^[*] ^(7-17)	27.0^(*) ^(13-33)
AMA	9.0 (7-11)	9.5 (6-14)	15.5<*> (11-24)
*LDH*^b^			
Control	15.0 (11-20)	18.5 (13-26)	40.5^[*] ^(11-59)
AMA	18.0 (10-23)	22.0 (16-31)	64.5^(*) ^(46-87)

*n = 6*/each treatment sub-group.

^a^shown as mIU/ml/10^5 ^cells/d. ^b^LDH activity as IU/10^5 ^cells/d at 37°C.

^(*)^P < 0.05 compared with any other group; ^[*]^P < 0.05 compared with other two sub-groups in control treatment group; ^<*>^P < 0.05 compared with other two sub-groups in AMA treatment group.

Cytokeratin 18 neo-epitope level in cells and leakage of LDH in media were higher (P < 0.05) at 96 h of culture as compared to 24 h and 48 h with no change between 24 h and 48 h (Figures 3M-O, 4; Tables 2 and 3). The profiles of TUNEL assays also revealed a detectable increase (P < 0.05) in the number of TUNEL-positive cells at 96 h as compared to 24 h and 48 h, with no change observed between 24 h and 48 h (Table 2).

### Effect of AMA

Administration of AMA at a concentration of 1000 ng/ml resulted in reduced numbers of cells and cell aggregates (Figures 2D-F) along with a tendency of decreased βhCG and hPL synthesis and secretion at each time point, most markedly (P < 0.05) at 96 h (Figures 3D-F, J-L, 4; Tables 2 and 3). Also, administration of AMA resulted in increased amount of cytokeratin 18 neo-epitope formation (Figures 3P-R, 4; Tables 2 and 3) and the number of TUNEL-positive cells along with higher degree of LDH leakage into the conditioned medium with marked (P < 0.05) effect being observed at 96 h (Table 3).

## Discussion

In the present study we could obtain an enriched population of cytokeratin 7 and βhCG positive and CD51 negative CTB from first trimester placental villi based on a method of sequential enzymatic treatment and plating the isolated cells on collagen I. It has been demonstrated earlier that cytokeratin 7- and βhCG-positive and CD51 (vitronectin receptor)-negative CTB were primarily villous in source, while cytokeratin 7-, βhCG- and CD51-positive CTB were derived from extravillous in source and invasive in nature [24,32]. Villous CTB in the present primary culture model displayed functional differentiation marked by increasing synthesis and secretion of hCG and hPL over time. In the present study, trophoblasts retained their biological functionality and formed aggregates of fused cells in primary culture on collagen [27]. However, we have not tested for syncytialization using staining for membrane markers such as E-cadherin or fodrin [33,34]; so we could not assess the numbers of syncytia in our cultures.

Previously, several dynamic processes related to CTB differentiation have been studied using primary cultures of human placental villous trophoblast cells as a model system [16,27,35]. It was earlier demonstrated that mononucleated CTB isolated from placental villi by trypsinization aggregated in a timed manner and fused to form syncytiotrophoblast (STB) that synthesized and secreted hCG and hPL [36,37]. Typically, CTB retrieved from first trimester placental villi have been shown to aggregate and start synthesizing hCG and hPL in culture by 3-days [27,35]. Similar results were obtained in the present study. A relatively high level of secretion of both hormones by 48 h and more markedly at 96 h along with marked degree of cell aggregration might be indicative of positive influence of hCG on CTB differentiation as well [38]. It is also apparent from the results of the present study that a higher number of cells underwent apoptosis and showed loss of viability as evident from higher level of cytokeratin 18 neo-epitope in cells, higher number of TUNEL-positive cells, and higher concentration of LDH leaked into the conditioned medium at 96 h as compared to 24 h and 48 h cultures. It is generally believed that these three parameters relate well with the apoptosis process and loss of cell viability [39]. The additional observation that the propidium iodide positive cells showing binding to annexin V increased at 96 h as compared with that at 24 h and 48 h (*data not shown*) also substantiated the conclusion [40].

We also observed that exposure of villous CTB to the cationic peptide AMA resulted in decreased production and secretion of βhCG and hPL along with increased degree cell apoptosis and loss of viability assessed from cytokeratin 18 neo-epitope level and TUNEL-positive nuclei containing cells, and LDH leakage into medium, respectively. These actions of AMA collectively might have resulted in observed pregnancy inhibition in monkeys [9,15].

The effect of AMA in attenuating the process of functional differentiation in terms of βhCG and hPL synthesis and secretion and that on cell viability in primary culture as observed in the present study should be considered against the background that villous trophoblasts follow two tightly regulated pathways of differentiation during placental development giving rise to differentiated CTB in anchoring villi and floating villi. In the anchoring villi, CTB aggregate into cell columns and invade maternal decidua, while in the floating villi, CTB differentiate into STB [41]. Thus, the present results document that AMA inhibited only the pathway of CTB differentiation into STB *in vitro*.

In a previous dose finding study, we observed that first trimester human placental villous CTB maintained in three dimensional culture subjected to AMA (1000 ng/ml) treatment in the presence of serum (10%, v/v) did not result in any significant change in their mitochondrial viability and hCG secretion, however, with detectable reduction in invasion efficiency at 48 h as compared to the control treatment (25). In the present study, same concentration of AMA in serum-free medium resulted in attenuation of differentiation, enhancement in apoptosis and loss of viability in early placental villi trophoblast cells at 96 h *in vitro*. Thus, it appears that serum factors might render a potential protection to cytotoxic action of AMA on early placental villous CTB. The possibility that serum factors may provide protection to mammalian cells from AMA mediated cytotoxicity has also been indicated earlier by others [3,42].

The underlying mechanism of the observed AMA mediated intervention of trophoblast function in the present study is not known, however, it appears that two potential mechanisms may be examined in this regard. Firstly, AMA might engage anionic domain in the cell membrane of CTB during the process of syncytialization and thereby resulted in functional inadequacy and loss of viability as we proposed in the background section based on previous reports [8,43]. It would indeed be of interest to directly examine the involvement of AMA with CTB cell membrane using high resolution time lapse photomicrography with suitable fluorochrome labels. The model used in the present study however did not allow this type of investigation. Secondly, AMA might act through calcium mediated process as suggested in studies of magainin 2 mediated histamine release [44] and caspase-independent apoptosis [45]. Indeed there is evidence to support that STB of first trimester placenta express transient receptor potential (TRPC) homologues that are involved in store-operated calcium entry [46]. Therefore it will be interesting to study calcium imaging in CTB treated with and without AMA *in vitro*. In this context, it is notable that our pilot experiments with isolated human endometrial stromal cells grown on collagen biomatrix failed to document any marked change in the cellular behavior and viability following AMA administration, unlike isolated CTB *in vitro *as observed in the present study. Thus, it indicates that AMA mediated a specific effect on the differentiation dynamics and viability of first trimester placental villous CTB maintained in primary culture on collagen. Further experiments are needed to investigate the anti-nidatory risk factors of AMP, *vis-à-vis*, their potential application to combat reproductive tract infection saving pregnancy based on new therapeutic antibiotic approach [47]. It is interesting to note in this context that AMA is an effective broad-spectrum, non-specific antimicrobial agent that also inhibits Herpes simplex virus types 1 and 2 also [2-6,48].

## Conclusions

The antimicrobial cationic peptide AMA attenuates differentiation and enhances apoptosis in primary villous CTB isolated from first trimester placenta. Further studies to delineate the effect of AMA on early stage placental trophoblast cells may give important leads towards understanding the anti-implantation risk factors in new therapeutic antibiotic approaches.

## Competing interests

The authors declare that they have no competing interests.

## Authors' contributions

JS, BH and DG contributed to the conception, designing, acquisition, analysis and interpretation of data and the drafting process of the manuscript. MAK contributed in performing experiments, data acquisition and analysis. All authors read and approved the final manuscript.

## References

[B1] Gottler LM Ramamoorthy A Structure, membrane orientation, mechanism, and function of pexiganan--a highly potent antimicrobial peptide designed from magainin Biochim Biophys Acta 2009 1788 1680 1686 10.1016/j.bbamem.2008.10.009 19010301 PMC2726618

[B2] Zaiou M Multifunctional antimicrobial peptides: therapeutic targets in several human diseases J Mol Med 2007 85 317 329 10.1007/s00109-006-0143-4 17216206

[B3] Hancock REW Sahl HG Antimicrobial and host-defense peptides as new anti-infective therapeutic strategies Nature Biotechnol 2006 24 1551 1557 10.1038/nbt1267 17160061

[B4] Zasloff M Antimicrobial peptides of multicellular organisms Nature 2002 415 389 395 10.1038/415389a 11807545

[B5] Hancock REW Lehrer R Cationic peptides: a new source of antibiotics Trends Biotechnol 1998 16 82 88 10.1016/S0167-7799(97)01156-6 9487736

[B6] Shai Y From innate immunity to de-novo designed antimicrobial peptides Current Pharmaceut Design 2002 8 715 725 10.2174/1381612023395367 11945167

[B7] Matsuzaki K Murase O Fujii N Miyajima K An antimicrobial peptide, magainin 2, induced rapid flip-flop of phospholipids coupled with pore formation and peptide translocation Biochemistry 1996 35 11361 11368 10.1021/bi960016v 8784191

[B8] Huppertz B Frank HG Reister F Kingdom JC Korr H Kaufmann P Apoptosis cascade progresses during turnover of human trophoblast: analysis of villous cytotrophoblast and syncytial fragments *in vitro* Lab Invest 1999 79 1687 1702 10616217

[B9] Dhawan L Ghosh D Lalitkumar PGL Sharma DN Lasley BL Overstreet JW Sengupta J Anti-nidatory effect of vaginally administered (Ala^8,1,18^)-magainin II amide in the rhesus monkey Contraception 2000 62 39 43 10.1016/S0010-7824(00)00134-7 11024227

[B10] Sandgren S Wittrup A Cheng F Jonsson M Eklund E Busch S Belting M The human antimicrobial peptide LL-37 transfers extracellular DNA plasmid to the nuclear compartment of mammalian cells via lipid rafts and proteoglycan-dependent endocytosis J Biol Chem 2004 279 17951 17956 10.1074/jbc.M311440200 14963039

[B11] Zasloff M Brian M Chen HC Antimicrobial activity of synthetic magainin peptides and several analogues Proc Natl Acad Sci (USA) 1988 85 910 913 10.1073/pnas.85.3.910 PMC279666 3277183

[B12] Chen HC Brown JH Morell JL Huang CM Synthetic magainin analogues with improved antimicrobial activity FEBS Lett 1988 236 462 466 10.1016/0014-5793(88)80077-2 3410055

[B13] Zasloff M Magainins, a class of antimicrobial peptides from Xenopus skin: isolation, characterization of two active forms, and partial cDNA sequence of a precursor Proc Natl Acad Sci (USA) 1987 84 5449 5453 10.1073/pnas.84.15.5449 PMC298875 3299384

[B14] Bevins CL Zasloff M Peptides from frog skin Ann Rev Biochem 1990 59 395 414 10.1146/annurev.bi.59.070190.002143 2197979

[B15] Ghosh D Dhawan L Lalitkumar PGL Wong V Rosario JF Hendrickx AG Sengupta J Effect of vaginally administered (Ala^8,13,18^)-magainin II amide on the morphology of implantation stage endometrium in the rhesus monkey (*Macaca mulatta*) Contraception 2001 63 335 342 10.1016/S0010-7824(01)00211-6 11672557

[B16] Ringler GE Strauss JF III In-vitro systems for the study of human placental endocrine function Endocr Rev 1990 11 105 123 10.1210/edrv-11-1-105 2180684

[B17] Hoshina M Hussa R Pattillo R Camel HM Boime I The role of trophoblast differentiation in the control of the hCG and hPL genes Adv Exp Med Biol 1984 176 299 312 6496214 10.1007/978-1-4684-4811-5_17

[B18] Kato Y Braunstein GD Purified first and third trimester placental trophoblasts differ in *in vitro *hormone secretion J Clin Endcrinol Metab 1990 70 1187 1192 10.1210/jcem-70-4-1187 2318939

[B19] Leers MP Kolgen W Bjorklund V Bergman T Tribbick G Persson B Bjorklund P Ramaekers FC Bjorklund B Nap M Jornvall H Schutte B Immunocytochemical detection and mapping of a cytokeratin 8 neo-epitope exposed during early apoptosis J Pathol 1999 187 567 572 10.1002/(SICI)1096-9896(199904)187:5<567::AID-PATH288>3.0.CO;2-J 10398123

[B20] Negoescu A Guillermet C Lorimier P Brambilla E Labat-Moleur F Importance of DNA fragmentation in apoptosis with regard to TUNEL specificity Biomed Pharmacother 1998 52 252 258 10.1016/S0753-3322(98)80010-3 9755824

[B21] Fotakis G Timbrell JA In-vitro cytotoxicity assays: Comparison of LDH, neutral red, MTT and protein assay in hepatoma cell lines following exposure to cadmium chloride Toxicol Lett 2006 160 171 177 10.1016/j.toxlet.2005.07.001 16111842

[B22] Kliman HJ Nestler JE Sermasi E Sanger JM Strauss JF III Purification, characterization, and *in vitro *differentiation of cytotrophoblasts from human term placentae Endocrinology 1986 118 1567 1582 10.1210/endo-118-4-1567 3512258

[B23] Bischof P Friedli E Martelli M Campana A Expression of extracellular matrix-degrading metalloproteinases by cultured human cytotrophoblast cells: effects of cell adhesion and immunopurification Am J Obstet Gynecol 1991 165 1791 1801 1750477 10.1016/0002-9378(91)90034-o

[B24] Tarrade A Lai Kuen R Malassiné A Tricottet V Blain P Vidaud M Evain-Brion D Characterization of human villous and extravillous trophoblasts isolated from first trimester placenta Lab Invest 2001 81 1199 1211 10.1038/labinvest.3780334 11555668

[B25] Lamba P Kar M Sengupta J Ghosh D Effect of (Ala^8,13,18^)-magainin II amide on human trophoblast cells *in vitro* Indian J Physiol Pharmacol 2005 49 27 38 15881856

[B26] Zhou WH Du MR Dong L Yu J Li DJ Chemokine CXCL12 promotes the cross-talk between trophoblasts and decidual stromal cells in human first-trimester pregnancy Hum Reprod 2008 23 2669 2679 10.1093/humrep/den308 18687671

[B27] Handwerger S Aronow B Dynamic changes in gene expression during human trophoblast differentiation Recent Prog Horm Res 2003 58 263 281 10.1210/rp.58.1.263 12795423

[B28] Gavrieli Y Sherman Y Ben-Sasson SA Identification of programmed cell death *in situ *via specific labeling of nuclear DNA fragmentation J Cell Biol 1992 119 493 501 10.1083/jcb.119.3.493 1400587 PMC2289665

[B29] Negoescu A Lorimier P Labat-Moleur F Drouet C Robert C Guillermet C Brambilla C Brambilla E *In situ *apoptotic cell labeling by TUNEL method: improvements and evaluation of cell preparation J Histochem Cytochem 1996 44 959 968 10.1177/44.9.8773561 8773561

[B30] Ghosh D Najwa AR Khan MA Sengupta J IGF2, IGF binding protein 1, and matrix metalloproteinases-2 and -9 in implantation-stage endometrium following immunoneutralization of vascular endothelial growth factor in the rhesus monkey Reproduction 2011 141 501 509 10.1530/REP-10-0475 21292726

[B31] Lum G Gambino SR A comparison of serum versus heparinized plasma for routine chemistry tests Am J Clin Pathol 1974 61 108 113 4809144 10.1093/ajcp/61.1.108

[B32] Aboagye-Mathiesen G Laugesen J Zdravkovic M Ebbesen P Isolation and characterization of human placental trophoblast subpopulations from first-trimester chorionic villi Clin Diagnost Lab Immunol 1996 3 14 22 10.1128/cdli.3.1.14-22.1996 PMC170241 8770498

[B33] Gauster M Siwetz M Orendi K Moser G Desoye G Huppertz B Caspases rather than calpains mediate remodelling of the fodrin skeleton during human placental trophoblast fusion Cell Death Differ 2010 17 336 345 10.1038/cdd.2009.133 19798107

[B34] Aplin JD Developmental cell biology of human villous trophoblast: current research problems Int J Dev Biol 2010 54 323 329 10.1387/ijdb.082759ja 19876840

[B35] Aronow B Richardson BD Handwerger S Microarray analysis of trophoblast differentiation: gene expression reprogramming in key gene function categories Physiol Genomics 2001 6 105 116 11459926 10.1152/physiolgenomics.2001.6.2.105

[B36] Jeschke U Richter DU Walzel H Bergemann C Mylonas I Sharma S Keil C Briese V Friese K Stimulation of hCG and inhibition of hPL in isolated human trophoblast cells *in vitro *by glycodelin A Arch Gynecol Obstet 2003 268 162 167 10.1007/s00404-002-0360-1 12942243

[B37] Handschuh K Guibourdenche J Tsatsaris V Guesnon M Lauendeau I Evain-Brion D Fournier T Human chorionic gonadotropin expression in human trophoblasts from early placenta: comparative study between villous and extravillous trophoblastic cells Placenta 2007 28 175 184 10.1016/j.placenta.2006.01.019 16584772

[B38] Shi QJ Lei ZM Rao CV Lin J Novel role of human chorionic goandotropin in differentiation of human cytotrophoblasts Endocrinology 1993 132 1387 1395 10.1210/en.132.3.1387 7679981

[B39] Johnson JE Methods for studying cell death and viability in primary neuronal cultures Methods Cell Biol 1995 46 243 276 full_text 7541885 10.1016/s0091-679x(08)61932-9

[B40] van Heerde WL Robert-Offerman S Dumont E Hofstra L Doevendans PA Smits JFM Daemen MJAP Reutelingsperger CPM Markers of apoptosis in cardiovascular tissues: focus on Annexin V Cardiovascul Res 2000 45 549 559 10.1016/S0008-6363(99)00396-X 10728376

[B41] Knofler M Critical growth factors and signaling pathways controlling human trophoblast invasion Int J Dev Biol 2010 54 269 280 10.1387/ijdb.082769mk 19876833 PMC2974212

[B42] McPhee JB Scott MG Hancock RE Design of host defence peptides for antimicrobial and immunity enhancing activities Comb Chem High Throughput Screen 2005 8 257 272 10.2174/1386207053764558 15892627

[B43] Huppertz B Kingdom JC Apoptosis in the trophoblast - role of apoptosis in placental morphogenesis J Soc Gynecol Investig 2004 11 353 362 10.1016/j.jsgi.2004.06.002 15350247

[B44] Hook WA Tsuji S Siraganian RP Magainin-2 releases histamine from rat mast cells Proc Soc Exp Biol Med 1990 193 50 55 1688467 10.3181/00379727-193-42989

[B45] Kulkarni MM McMaster WR Kamysz W McGwire BS Antimicrobial peptide-induced apoptotic death of leishmania results from calcium-dependent caspase-independent mitochondrial toxicity J Biol Chem 2009 284 15496 15504 10.1074/jbc.M809079200 19357081 PMC2708846

[B46] Clarson LH Roberts VHJ Hamark B Elliot AC Powell T Store-operated Ca^2+ ^entry in first trimester and term human placenta J Physiol 2003 550.2 515 528 10.1113/jphysiol.2003.044149 PMC2343039 12766233

[B47] Yedery RD Reddy KV Antimicrobial peptides as microbicidal contraceptives: prophecies for prophylactics - a mini review Eur J Contracept Reprod Health Care 2005 10 32 42 10.1080/13625180500035124 16036297

[B48] Albiol Matanic VC Castilla V Antiviral activity of antimicrobial cationic peptides against Junin virus and Herpes simplex virus Int J Antimicrob Agents 2004 23 382 389 10.1016/j.ijantimicag.2003.07.022 15081088

